# Association of Depression and Antidepressant Use With Driving Behaviors in Older Adults: A LongROAD Study

**DOI:** 10.1177/07334648241238313

**Published:** 2024-03-13

**Authors:** Chelsea A. Isom, Sara Baird, Marian E. Betz, Carolyn G. DiGuiseppi, David W. Eby, Guohua Li, Kelly C Lee, Lisa J. Molnar, Ryan Moran, David Strogatz, Linda Hill

**Affiliations:** 1 Herbert Wertheim School of Public Health and Longevity Science, 8784 University of California San Diego, San Diego, CA, USA; 2 Department of Emergency Medicine, 12225 University of Colorado School of Medicine, Aurora, CO, USA; 3 VA Eastern Colorado Geriatric Research Education and Clinical Center, Aurora, CO, USA; 4 Department of Epidemiology, Colorado School of Public Health, 129263 University of Colorado Anschutz Medical Campus, Aurora, CO, USA; 5 62202 University of Michigan Transportation Research Institute, Ann Arbor, MI, USA; 6 Department of Epidemiology, 33638 Mailman School of Public Health, Columbia University, New York, NY, USA; 7 Department of Anesthesiology, Vagelos College of Physicians and Surgeons, Columbia University, New York, NY, USA; 8 San Diego Skaggs School of Pharmacy and Pharmaceutical Sciences, 8784 University of California, La Jolla, CA, USA; 9 Bassett Healthcare Network, 138422 Bassett Research Institute, Cooperstown, NY, USA

**Keywords:** depression, driving, medication

## Abstract

Older adults aged 70 and older who drive have higher crash death rates per mile driven compared to middle aged (35–54 years) adults who drive in the US. Prior studies have found that depression and or antidepressant medication use in older adults are associated with an increase in the vehicular crash rate. Using data from the prospective multi-site AAA Longitudinal Research on Aging Drivers Study, this analysis examined the independent and interdependent associations of self-reported depression and antidepressant use with driving behaviors that can increase motor vehicle crash risk such as hard braking, speeding, and night-time driving in adults over age 65. Of the 2951 participants, 6.4% reported having depression and 21.9% were on an antidepressant medication. Correcting for age, race, gender, and education level, participants on an antidepressant had increased hard braking events (1.22 [1.10–1.34]) but self-reported depression alone was not associated with changes in driving behaviors.

**What this paper adds**

• Older drivers on an antidepressant medication drove fewer miles and had increased risk for hard braking, which has been associated with increased risk of rear end crashes.
• In the study population of older community dwelling driving adults, we found that 6.4% had active depression symptoms and 21.9% were on at least one anti-depressant.
• Night-time driving was not impacted by depression or antidepressant use and represented only 7% of driving time in this cohort.

**Application of study findings**

• As the number of older drivers continues to increase, it is important to know what medications can impact their driving, in order to offer education about the risks.
• Antidepressants are associated with an increased risk of hard braking, after starting an antidepressant clinicians could screen for increased braking events to determine if there has been a change in the patients’ driving behaviors specific to antidepressant use.
• These results show that in the US older adult drivers do most of their driving during the day, indicating that is their preferred driving time, when scheduling visit or procedures institutions could recommend appointments that would fit within this period.

## Introduction

In 2019, there were over 6 million motor vehicle crashes (MVC) in the United States (US), leading to 33,244 fatalities (Center for Disease Control and Prevention, National Statistics, 2023; Insurance Institute for Highway Safety, 2021). Older adults aged 70 and older who drive have higher crash death rates per mile driven compared to middle aged (35–54 years) adults who drive in the US (Center for Disease Control and Prevention. Transportation and Elderly Drivers, 2023). More than 8000 adults over the age of 65 were killed in traffic crashes in 2019 (Center for Disease Control and Prevention. Transportation and Elderly Drivers, 2023). In 2018, over 45 million adults over age 65 held driver licenses in the US, which is a 60% increase since 2000 (Center for Disease Control and Prevention. Transportation and Elderly Drivers, 2023). By 2050, as the US population ages, nearly 1 in 4 drivers are expected to be over the age of 65 (Ng et al., 2020).

Impaired reaction times, decreased muscle strength, reduced vision, and slowed cognition can all occur as a byproduct of the aging process and/or from medical conditions and medications, which may contribute to decreased driving performance and an increased risk of crashing (Ng et al., 2020). Multiple studies have shown that depression and antidepressant use can impair driving performance (Aduen et al., 2015; Babulal et al., 2018; Bulmash et al., 2006; Cameron & Rapoport, 2016; Dassanayake et al., 2011; Hill et al., 2017; Narredo et al., 2018; Ng et al., 2020; Ragland et al., 2005). A systematic review that examined adults over 55 years found that antidepressant use was associated with an increased risk of collisions (Cameron & Rapoport, 2016). A prospective cohort out of France found that adults (mean age 60) who had self-reported depression were at increased risk of crash even when they self-limited their driving behavior over time, decreasing miles driven (Narredo et al., 2018). A meta-analysis showed that both depression and antidepressant use or the combination of the two can contribute to unsafe driving (Hill et al., 2017).

To date, studies have focused on the outcomes of crash or injury and have not evaluated what driving behaviors may be affected by depression and antidepressant use (Aduen et al., 2015; Babulal et al., 2018; Cameron & Rapoport, 2016; Narredo et al., 2018; Ng et al., 2020). The goal of this analysis was to use naturalistic driving to examine the independent and interdependent associations of self-reported depression and antidepressant use with driving behaviors that can increase MVC risk such as hard braking, speeding, night-time driving, and total number of miles driven (Eby et al., 2019; O’Connor et al., 2012; Ross et al., 2009).

## Methods

The AAA Longitudinal Research on Aging Drivers (LongROAD) study is a multi-site longitudinal prospective cohort study of older adults who drive in the US, the methods and protocols have been previously published but will briefly be reviewed, focusing on key elements relevant to this analysis (Li et al., 2017). The AAA LongROAD study enrolled community dwelling adults between the ages of 65–79 who operated a vehicle, from July 2015 to March 2017. Drivers were excluded if they had diagnoses thought to contribute to unsafe driving (dementia, Parkinson, blindness, memory loss, late-stage cancers, or end stage diseases). The study was conducted at five sites across the US, (Ann Arbor MI, Baltimore MD, Cooperstown NY, Denver CO, and San Diego CA), each with approval from their institutional review board. Baseline measures collected on all participants included standardized cognitive tests, psychological tests, and physical functioning, along with driving measures. Medication review was done via “brown bag” review, where the participants brought all active prescription, over the counter, and vitamin/supplements to an in-person visit; these were then recorded into a centralized database. Each medication was coded using the American Hospital Formulary Service (AHFS) Pharmacologic Therapeutic Classification system (American Society of Health-System Pharmacists Pharmacologic Therapeutics Classification System, 2023; Li et al., 2017). Driving behavior was captured with a GPS/accelerometer device called a “Datalogger” which was installed in each participant’s vehicle. The device recorded data that were later processed to show the vehicle’s trip length, time, location, speed, and hard decelerations. Data from the first 12 months of each participant’s Datalogger were included for analysis. Participants were excluded from this analysis if they declined to have the datalogger installed or it was not compatible with their vehicle (*n* = 39).

Participants were categorized as having self-reported depression based on the Patient-Reported Outcomes Measurement Information System (PROMIS^®^) survey administered at baseline (Schalet et al., 2016). The PROMIS depression scale is a validated tool to measure current depression symptoms, with a standard cut off for symptoms of depression of 55 (Health Measures, 2023; Schalet et al., 2016). Participants whose baseline medication review included the class code for antidepressant, (AHFS tier 25:16.04), were considered to be on an antidepressant and coded as a binary variable of “yes” or “no” (American Society of Health-System Pharmacists Pharmacologic Therapeutics Classification System, 2023). This antidepressant group included: monoamine oxidase inhibitors, selective serotonin and norepinephrine reuptake inhibitors, selective serotonin-reuptake inhibitors, serotonin modulators, tricyclics, and miscellaneous.

Participants were categorized based on their use of antidepressant medication and depression status as a PROMIS score greater than or equal to 55. The four groups included: self-reported depression and current use of antidepressant (D+M+), self-reported depression and no current use of antidepressant (D+M−), no self-reported depression and current use of antidepressant (D−M+), and no self-reported depression and no current use of antidepressant (D−M−), which served as the reference group.

To determine the association of self-reported depression and antidepressant use with driving behaviors, we measured speeding events, hard braking events, night-time driving, and total miles driven over a 12-month period. As this was a rolling enrollment, the 12-month period started when the participant had the data logger installed in their vehicle. A speeding event was defined as traveling at 80 mph or greater for 7 seconds or more. A hard braking event was defined as a deceleration of 3.5G or more. Night-time driving was defined as a trip where 80% of the trip occurred during the night-time, defined as a solar angle of greater than 96°.

Participant age at enrollment was categorized as 65–69, 70–74, and 75–79 years and additional demographics included gender, and racial identity, categorized as shown in Table 2. Volunteer and working status were included as it may impact propensity or need to drive. Each participant was asked if they worked or volunteered outside the home, which was recorded as yes, no, or declined to answer. Education level was grouped into four categories: high school or less, some college (no degree), associates or bachelors, and advanced degree.

Demographics were analyzed using the Chi-squared method, with significance level defined as *p*=< 0.05 (Table 2). Each driving variable was analyzed based on self-reported depression and antidepressant use alone using the Chi-squared or *t*-test when appropriate (Table 3). The relationship between antidepressant class and depression status was evaluated to determine if antidepressant class differed based on depression status. The frequency of speeding events and hard braking events were calculated as number of events per 1000 miles driven, while the outcome for night-time driving was defined as the percentage of all trips that were driven in the night-time. We utilized a generalized linear mixed model with random intercepts to account for repeated measures to evaluate total miles driven, and a Poisson regression model with random intercepts to account for repeated measure and offset for miles driven per year to evaluate speeding events, hard braking events, and trips at night. Self-reported depression was not statistically significant in our models for hard braking events; therefore, we only present antidepressant use for this variable. All models were adjusted for the following potential confounders, age, race, gender, and level of education. Analysis was done using SAS version 9.9 and STATA version 17.0.

## Results

Of the 2990 participants in the baseline LongROAD cohort, 2951 participants (99%) were included in this analysis. The participants were 53.0% female, and 85.5% White non-Hispanic. At baseline, 6.4% of the participants met the PROMIS cutoff for self-reported depression, and 21.9% of the participants were on an antidepressant medication. Of participants on an antidepressant medication, 18.5% were on a selective serotonin and norepinephrine reuptake inhibitor, 50.7% were on a selective serotonin-reuptake inhibitor, 8.4% were on a serotonin modulator, and 8.8% were on a tricyclic or other norepinephrine reuptake inhibitor and 13.6% were on another antidepressant (Table 1). There was no statistically significant difference between subtype of antidepressant medication and depression status (*p* = .411).

**Table 1.** List of Antidepressant Categories.

Name of Antidepressant Category	AHFS Code	List of Antidepressants in Category	Number of Participants on Medication^a^
Serotonin and norepinephrine reuptake inhibitors	28:16:04:16	desvenlafaxine, duloxetine, levomilnacipran, venlafaxine	119 (18.5%)
Selective serotonin reuptake inhibitors	28:16:04:20	citalopram, escitalopram, fluoxetine, paroxetine, sertraline	327 (50.7%)
Serotonin modulators	26:16:04:24	nefazodone, trazadone, vilazodone, vortioxetine	54 (8.4%)
Tricyclics and other norepinephrine reuptake inhibitors	26:16:04:28	amitriptyline, amoxapine, clomipramine, desipramine, doxepin, imipramine, maprotiline, nortriptyline, protriptyline	57 (8.8%)
Miscellaneous	28:16:04:92	bupropion, mirtazapine	88 (13.6%)

Presented is a list of all medication in each American Hospital Formulary Service (AHFS) category, the corresponding code number, and percentage of each group in the sample.

^a^Percentage is the count of a specific antidepressants over the total number of antidepressants not total number of participants.

Participants drove an average of 739 ± 431 miles a month or 8869 ± 5174 miles a year and an average of only 6.2 ± 3.1 miles per trip over the entire year. Most drivers limited their driving to daytime with a mean percentage of trips during the day of 93.1 ± 5.2%. Participants on average drove 22 ± 4.8 days a month. Speeding (>80 mph for 7 or more seconds) was not common, averaging 7.8 events per 1000 miles driven.

There were statistically significant differences among depression status and antidepressant use categories by age group (*p* = .007), gender (*p* < .001), and race/ethnicity (*p* = .006) (Table 2). Of participants with self-reported depression and antidepressant use (D+M+), 57.5% were in the youngest age category, whereas 39.9% of the reference group (D−M−) were in the youngest category. Among participants with self-reported depression (D+), 3.5% of those taking an antidepressant (M+) were Black non-Hispanic, whereas among those not taking antidepressant (M−) 9.8% were Black non-Hispanic. Of those without self-reported depression, (D−), 68.2% of those taking an antidepressant (M+) were women, while 48.4% of those not taking an antidepressant (D−), were women.

**Table 2.** Participant Demographics.

	D−M−n = 2204	D−M+n = 558	D+M−n = 102	D+M+n = 87	*p*-Value
Age categories					.007
65–69	879 (39.9%)	251 (45.0%)	48 (47.1%)	50 (57.5%)	
70–74	778 (35.3%)	190 (34.1%)	34 (33.3%)	19 (21.8%)	
75–79	547 (24.8%)	117 (20.9%)	20 (19.6%)	18 (20.7%)	
Gender					<.001
Male	1138 (51.6%)	172 (30.8)	41 (40.2%)	32 (36.8%)	
Female	1066 (48.4%)	386 (68.2%)	61 (59.8%)	55 (63.2%)	
Race					.006
White, non-Hispanic	1862 (84.5%)	504 (90.3%)	81 (79.4%)	77 (88.5%)	
Black, non-Hispanic	166 (7.5%)	31 (5.6%)	10 (9.8%)	3 (3.5%)	
Asian	55 (2.5%)	2 (0.4%)	5 (4.9%)	2 (2.3%)	
Other^a^	121 (5.5%)	21 (3.8%)	6 (5.9%)	5 (5.8%)	
Marital status					<.001
Married or living with a partner	1508 (68.9%)	336 (60.9%)	60 (58.9%)	46 (52.9%)	
Single	675 (30.9%)	216 (39.1%)	41 (40.2)	41 (47.1%)	
Declined to answer	5 (0.2%)	0	1 (0.9%)	0	
Volunteering					<.001
No	1154 (52.4%)	317 (56.8%)	69 (67.7%)	62 (71.3%)	
Yes	1036 (47.0%)	240 (43.0%)	32 (31.4%)	25 (28.7%)	
Decline to answer	14 (0.6%)	1 (0.2%)	1 (0.9%)	0	
Working					.012
No	1492 (67.7%)	420 (75.3%)	77 (75.5%)	67 (77.0%)	
Yes	710 (32.2%)	138 (24.7%)	25 (24.5)	20 (23.0%)	
Declined to answer	1 (0.1%)	0	0	0	
Education					.001
High school or less	226 (10.3%)	74 (13.3%)	19 (18.8%)	11 (12.7%)	
Some college (no degree)	365 (16.6%)	109 (19.5%)	21 (20.8%)	23 (26.4%)	
Associates or bachelors	659 (30.0%)	169 (30.3%)	27 (26.7%)	30 (34.5%)	
Advanced degree	946 (43.1%)	206 (36.9%)	34 (33.7%)	23 (26.4%)	

Patient demographics and social characteristics at baseline entrance into the study. Participants were divided into 4 categories based on depression status and antidepressant medication use. D = Depression M = antidepressant medication, + presence of condition or medication, - absence of condition or medication.

^a^Other included: American Indian, Alaskan Native, Native Hawaiian, Pacific Islander, Hispanic, other non-Hispanic, and decline to answer.

Participants with self-reported depression (D+) drove fewer total miles per year (*p* = .023) and fewer miles per driving day than those without depression (D−) (*p* = .006) (Table 3). Antidepressant (M+) use was also associated with driving fewer miles per driving day (*p* < .001) (Table 3). Neither depression (D+) nor antidepressant (M+) use was associated with speeding events (*p* = .6 and *p* = .9). Those on antidepressant (M+) medication had fewer trips at night than those not on an antidepressant (M+) (*p* = .02).

**Table 3.** Unadjusted Driving Behaviors.

	Miles Driven per Day^a^	Speeding Events per 1000 Miles^b^	Hard Braking Events per 100 Miles^c^	NIGHT-TIME Trips^d^
No depression (D−)	34	7.8	5.3	6.8
Depression (D+)	31*	8.2	5.4	6.3
No antidepressant (M−)	35	7.5	6.1	6.8
Antidepressant (M+)	31**	8.8	6.2	6.2**

Mean driving behaviors in those with and without self-reported depression or antidepressant medication use. *p < .05 when compared between No Depression and Depression groups. **p < .05 when compared between No Antidepressant and Antidepressant groups.

^a^Mean number of miles driven in a year averaged over the number of days driven.

^b^Mean number of speeding events per 1000 miles driven.

^c^Mean number of hard braking events per 1000 miles driven.

^d^Mean percentage of trips at night.

After accounting for age, race, gender, and education level, the number of miles driven per year was still statistically different the groups (Table 4). The model compared each of the three conditions/medication groups to the reference group (D−M−), meaning negative miles indicate a group drove fewer miles than the reference group and positive miles indicates a group drove more miles than the reference group. There was no difference in miles driven between the reference group and those with self-reported depression but not on an antidepressant (D+M−). However, those who were on an antidepressant without self-reported depression (D−M+) drove fewer miles than the reference group (−1577 miles; CI -2012 to −1123). Participants with both self-reported depression and antidepressant medication (D+M+) drove fewer total miles than any other group (−2903 miles; CI −3679 to −2129).

**Table 4.** Adjusted Miles Driven per Year.

Characteristic	Miles per Year	95% CI
D−M−	Reference	
D+M−	50	−937 to 1037
D−M+	−1577	−2012 to −1123*
D+M+	−2903	−3679 to −2129*
Age 65–69	Reference	
Age 70–74	−311	−789 to 167
Age 75–79	−1938	−2374 to −1502*
Race white non-Hispanic	Reference	
Race black non-Hispanic	−596	−1294 to 103
Race Asian	−1964	−3074 to – 853*
Race other	−1064	−1772 to −356*
Male	Reference	
Female	−1279	−1695 to −864*
High school or less	Reference	
Some college	924	209 to 1640*
Associates/bachelor’s degree	402	−217 to 1020
Advanced degree	514	−100 to 1128

A generalized linear mixed model with random intercepts was used to account for repeated measures to evaluate total miles driven which corrected for self-reported depression, antidepressant use and demographic criteria. The presence of * indicates statistically significant.

Hard braking events were statistically more likely for those on antidepressant (M+) (RR 1.22, CI: 1.1–1.34), after accounting for age, race, gender, and education (Tables 4 and 5). There were no statistically significant differences in rates of speeding events and percentage of night-time trips between the self-reported depression and antidepressant groups in the corrected models (data not presented). There was no statistical association between hard braking events and type of antidepressant (*p* = .15).

**Table 5.** Adjusted Hard Breaking Events.

Characteristic	Rate Ratio, Hard Braking	95% CI
No antidepressant	1.0	
Antidepressant	1.22	1.10–1.34*
Age 65–69	1.0	
Age 70–74	1.06	0.86–1.30
Age 75–79	1.09	0.8–1.48
Race white, non-Hispanic	1.0	
Race black non-Hispanic	1.71	1.27–2.32*
Race Asian	2.24	1.43–3.52*
Race other	2.93	1.60–5.36*
Male	1.0	
Female	1.21	1.02–1.45*
High school or less	1.0	
Some college	1.34	0.95–1.91
Associates/bachelor’s degree	1.56	0.92–2.64
Advanced degree	1.81	0.89–3.65

A Poisson regression model with random intercepts was used to account for repeated measures and offset for hard braking events associated with antidepressant use. Depression was not significant and not included in the model. The presence of * indicates statistical significance.

## Discussion

This study examined how antidepressant use and self-reported depression impact driving behaviors in older driving adults in the United States. We found that antidepressant use, and self-reported depression were significantly associated with hard braking events and fewer miles driven. Those with self-reported depression did not have a reduction in the total miles driven unless they were also taking an antidepressant. Neither self-reported depression nor antidepressant use was associated with speeding events or difference in night-time driving..

The prevalence of self-reported depression and antidepressant use found in the study population mirrors the expected prevalence in older community-dwelling residents. The Centers for Disease Control and Prevention estimates the prevalence of depression to be 1%–5% in older adults; 6.4% of our participants were identified as having self-reported depression (Center for Disease Control and Prevention. Depression is Not a Normal Part of Growing Older, 2023). Antidepressant use was documented in 21.8% of the participants, similar to the reported 19% of those aged 60 and older using an antidepressant in the 2018 National Health and Nutrition Examination Study (Brody & Gu, 2023). In 2022, drivers 65 years and older drove an average of 7646 miles (Guo et al., 2010). Our drivers averaged 8869 a year which is slightly higher; this may be due to our community-dwelling cohort being healthier than the average population (Federal Highway Administration, 2022). The cohort did 93% of their driving during daylight, which is consistent with studies that show older adults exhibit self-regulatory behavior, by restricting night-time driving and total miles driven (Beck et al., 2022; Ross et al., 2009).

A systematic review of older drivers found that antidepressant use increased the risk of MVCs, and our study results support this finding, in that antidepressant use increased the number of hard braking events (Cameron & Rapoport, 2016). Studies show that near crash behaviors (sudden maneuvers that prevent a crash) are similar to crashes and specifically hard braking has been shown to correlate with increased risk for crash (Eby et al., 2019; Guo et al., 2010; O’Connor et al., 2006). Studies have hypothesized that hard braking may indicate an inability to accurately assess spatial distances, which can lead to crashes (Keay et al., 2013).

A study in France showed that drivers with depression drive less than those without Narredo et al. (2018). In our study, participants on antidepressants drove fewer miles than those not on antidepressants, and those with self-reported depression symptoms and on an antidepressant drove the least total number of miles of all study groups. As the previous study did not look at medications, it is unclear if drivers with depression were on antidepressants.

While several studies have shown that depression increases crash risk and hazardous driving, we did not find any correlation between self-reported depression and hard braking or speeding events after adjusting for age, race, gender, and education (Babulal et al., 2018; Hill et al., 2017; Narredo et al., 2018). This may be because of differences in how depression was defined; these studies used patient self-reports, DSM criteria or national records. Several of the depression studies did not look at medication use, just at depression, which makes it hard to decipher whether depression symptoms, antidepressant use, or both were driving the association with crash risk. Our study used the PROMIS survey which assessed current depression symptoms instead of using medical diagnosis documented by a medical record. This method may have under-captured participants with depression diagnoses who did not report symptoms at the time of the test administration. On the other hand, by assessing current symptoms with a validated tool, we were able to include those experiencing depression symptoms who may not have received a formal diagnosis.

A limitation to the study is that participants may drive more than one vehicle, and driving behaviors would not have been captured in secondary vehicles. However, one of the LongROAD study inclusion criteria was that participants consent to drive only one vehicle at least 80% of the time. Speeding events, defined as driving >80 mph for 7 seconds or longer, can capture risky driving behavior. While this is important, this measure does not capture traveling above the posted speed limit. Further studies are needed to determine if depression or antidepressant use affect driving over the speed limit. Our study grouped all antidepressants into one category regardless of pharmacologic properties. While we found no difference between antidepressant subgroups and depression status, we recognize that antidepressants may have variable effects on driving behavior due to pharmacologic properties, such as anticholinergic effects, as well as patient’s genetic makeup that may influence metabolism. We are unable to differentiate these effects based on the current data. We acknowledge that mental health issues and medication administration, especially in older adults, is a complex issue that should be a shared medical decision between the patient and their healthcare provider. Finally, while driving behavior was captured longitudinally, we juxtaposed this with a single baseline assessment of medication use and depression symptoms.

Strengths of this study include the use of the brown bag method to verify each participant’s medication and the use of objective driving data collection. For medication reconciliation, brown bag review is preferred to relying on medical records, which can be out of date, incomplete or inaccurate. The datalogger in each vehicle was able to longitudinally track our participant’s actual driving behavior instead of relying on self-reported driving behaviors that may be affected by recall bias. The study was also designed to evaluate the role of depression and antidepressant medication independently to account for the individual impact of the disease itself and medication on driving behaviors.

In conclusion, in this study, we found that antidepressant use was associated with increased hard braking events and decreased total driving, however, further study is needed to further evaluate this complex relationship between driving and antidepressant medication.
